# Computerized Automated Quantification of Subcutaneous and Visceral Adipose Tissue From Computed Tomography Scans: Development and Validation Study

**DOI:** 10.2196/medinform.4923

**Published:** 2016-02-04

**Authors:** Young Jae Kim, Ji Won Park, Jong Wan Kim, Chan-Soo Park, John Paul S Gonzalez, Seung Hyun Lee, Kwang Gi Kim, Jae Hwan Oh

**Affiliations:** ^1^Biomedical Engineering BranchDivision of Convergence TechnologyResearch Institute, National Cancer CenterGoyangRepublic Of Korea; ^2^Department of Plazma Bio DisplayKwangWoon UniversitySeoulRepublic Of Korea; ^3^Department of SurgerySeoul National University College of MedicineSeoulRepublic Of Korea; ^4^Cancer Research InstituteSeoul National UniversitySeoulRepublic Of Korea; ^5^Colorectal Cancer Center, Seoul National University Cancer HospitalSeoulRepublic Of Korea; ^6^Department of SurgeryHallym Univerisy Dongtan Sacred Heart HospitalHwaseongRepublic Of Korea; ^7^Department of SurgeryPhilippine General HospitalUniversity of the Philippines ManilaManilaPhilippines; ^8^Center for Colorectal CancerNational Cancer CenterGoyangRepublic Of Korea

**Keywords:** obesity, visceral adipose tissue, subcutaneous adipose tissue, computed tomography, computer-assisted image analysis

## Abstract

**Background:**

Computed tomography (CT) is often viewed as one of the most accurate methods for measuring visceral adipose tissue (VAT). However, measuring VAT and subcutaneous adipose tissue (SAT) from CT is a time-consuming and tedious process. Thus, evaluating patients’ obesity levels during clinical trials using CT scans is both cumbersome and limiting.

**Objective:**

To describe an image-processing-based and automated method for measuring adipose tissue in the entire abdominal region.

**Methods:**

The method detects SAT and VAT levels using a separation mask based on muscles of the human body. The separation mask is the region that minimizes the unnecessary space between a closed path and muscle area. In addition, a correction mask, based on bones, corrects the error in VAT.

**Results:**

To validate the method, the volume of total adipose tissue (TAT), SAT, and VAT were measured for a total of 100 CTs using the automated method, and the results compared with those from manual measurements obtained by 2 experts. Dice’s similarity coefficients (DSCs) between the first manual measurement and the automated result for TAT, SAT, and VAT are 0.99, 0.98, and 0.97, respectively. The DSCs between the second manual measurement and the automated result for TAT, SAT, and VAT are 0.98, 0.98, and 0.97, respectively. Moreover, intraclass correlation coefficients (ICCs) between the automated method and the results of the manual measurements indicate high reliability as the ICCs for the items are all .99 (*P*<.001).

**Conclusions:**

The results described in this paper confirm the accuracy and reliability of the proposed method. The method is expected to be both convenient and useful in the clinical evaluation and study of obesity in patients who require SAT and VAT measurements.

##  Introduction

Obesity refers to the over-accumulation of adipose tissue (AT) in the body. Obesity can be caused by genetic and fat metabolism abnormalities, hypothyroidism, excessive nutritional intake, lack of exercise, and stress, and it is known to be a key factor in chronic diseases [1]. In recent studies, body fat distribution has been shown to pose a greater health risk than overall body fat, and among the different types of obesity based on specific categories of body fat distribution, abdominal obesity has been reported to pose the greatest risk [2]. Abdominal obesity increases the prevalence rate of metabolic syndrome accompanied by coronary artery diseases such as hypertension, diabetes, hyperlipidemia, and arteriosclerosis [3,4]. Because visceral adipose tissue (VAT), rather than subcutaneous adipose tissue (SAT), is recognized as the contributing factor in body insulin resistance, visceral abdominal obesity is viewed as the more clinically important type of abdominal obesity [5]. Furthermore, by causing physical pressure, the accumulation of heavy VAT can interrupt blood flow to abdominal organs and decrease organ function (eg, liver). As such, VAT can be even more deleterious than SAT [6]. The accurate evaluation and prevention of both SAT and VAT with quantitative fat measurements are thus important.

In recent clinical trials, diverse methods have been applied to assess obesity. For example, body mass index (BMI) can be used to evaluate obesity easily by height and weight measurements; however, VAT cannot be measured by this method. On the other hand, methods such as bioelectrical impedance analysis (BIA), magnetic resonance image (MRI), and computed tomography (CT) can acquire VAT measurements. BIA is a technique by which the percentage of body fat and the area of VAT are estimated by sending a weak- to high-frequency current through the body and measuring the bioelectrical impedance [7]. Recently, BIA has been widely used in diagnosing obesity owing to the simplicity of its measurement; many reports support its high-degree of accuracy in body fat mass measurements [8]. However, there are only a few reports on whether it can satisfactorily reflect the actual amount of VAT. On the other hand, CT can clearly distinguish AT from other tissues, and AT can be measured directly from the cross-sectional images of the tissue. In addition, CT has the advantage that SAT and VAT can also be directly measured [9]. Because the measurement method is cumbersome and poses a risk of radiation exposure, it is not in general use. However, the additional radiation exposure involved in measuring AT can be eliminated by using CT images obtained from health screening or other procedures, and by using low-dose CT, which is currently being used in the clinic. Finally, MRI, which is similar to CT, can be used to generate cross-sectional images and can measure tissue and VAT directly. Despite the advantage of no radiation exposure [10], MRI has clinical limitations because of the cumbersome measuring method, high-cost, and lengthy imaging times.

Among the various obesity evaluation methods, CT is considered the gold standard in clinical trials due to its high accuracy. However, AT has to be measured directly from the cross-sectional images, and when measuring VAT, the boundaries between SAT and VAT must be clearly defined. In other words, a substantial amount of time and effort have to be expended to analyze a single CT image. In abdominal obesity-related studies, such problems can act as limiting factors in analyzing large amounts of data or when analyzing a wide range of AT in the abdomen. Efforts have been made to minimize the time required to make measurements by calculating the level of abdominal obesity from a single CT image at the umbilical level (L4-5 vertebrae), which represents the entire abdominal fat area [11].

In this study, we attempt to solve these limiting factors by using an automated, computer image processing technique. By automating the image processing, the entire abdominal region can be measured in a relatively short time period. In addition, it eliminates subjectivity, enabling objective, quantitative, and reliable measurements to be made. An increasing number of studies have been conducted on the computerized, automated measurement of AT. For example, using a single CT image at the umbilical level, Bandekar et al proposed the use of the active shape model and fuzzy affinity-based automatic fat analysis to distinguish between SAT and VAT [12]. In the study, they compared the results with those obtained manually by experts, and they calculated and evaluated both accuracy and sensitivity. They determined the degree of accuracy for SAT and VAT to be 98.29% (SD 0.62%) and 97.66% (SD 0.98%), respectively [12]. Zhao et al also reported automated separation of SAT and VAT using pixel information obtained from radial movement at increments of 3 degrees from the center of the body from a single CT image at the umbilical level [13]. In that study, 9 subjects were tested and the differences between the automated and manual measurements in terms of SAT and VAT were 0.65% and 1.54%, respectively [13]. In another example, Kullberg et al used a histogram based on MRI images at the umbilical level range and constructed AT and SAT masks to isolate each AT [14]. An algorithm was applied to a total of 17 data values, using the manual measurements as the reference, and obtained true positive measurements for SAT and VAT, with high accuracy values of 96% (SD 2.3%) and 90% (SD 6.5%), respectively [14]. Although the studies mentioned above showed a high degree of accuracy, the verification data was minimal, and the allowed range of measurement was limited to a single image or restricted to the umbilical level only. In order to overcome these limitations, we propose a method for the automatic separation and measurement of SAT and VAT in the entire abdominal region using CT. By comparing a large amount of test data with manually measured results, the technical and clinical utility of the proposed method was verified and evaluated.

## Methods

### Study Dataset and Development Environment

This study was approved by the Institutional Review Board (IRB) of the National Cancer Center of Korea with a waiver of the requirement for patients’ informed consent (1210160-3).

In this study, abdominal CTs on 100 patients from the National Cancer Center of Korea were analyzed. Images were obtained using LightSpeed VCT (GE Healthcare) and Brilliance 64 (Phillips). For each CT scan, an image size of 512 × 512 pixels and slice thicknesses of 2.5 to 5.0 mm were used. We measured the abdominal area, which included the region from the diaphragm to coccyx and excluded the pelvic cavity. Here, Microsoft Visual Studio (Ver. 2005, Microsoft, Redmond, US) was used for the development of algorithms and software, and ITK (Ver. 3.14.0, Kitware, US) and VTK (Ver. 5.10.0, Kitware, US) were used as libraries. The SPSS package (Ver. 13, SPSS Inc., US) was used for statistical analysis.

### Manual Measurement

The manual measurements reported in this paper were taken directly by 2 experts using in-house developed software. The in-house software depicts the AT area using a brush to color the area directly on the CT image; SAT and VAT are distinguished by applying different colors to each. For the convenience of the user, only areas within the AT attenuation range of -30 to -190 Hounsfield units (HU) in CT and multi-planar reconstruction were considered [11,15]. The manual measurement results were used as the gold standard data in comparisons with the automated measurement results.

### Automatic Measurement

**Figure 1:**
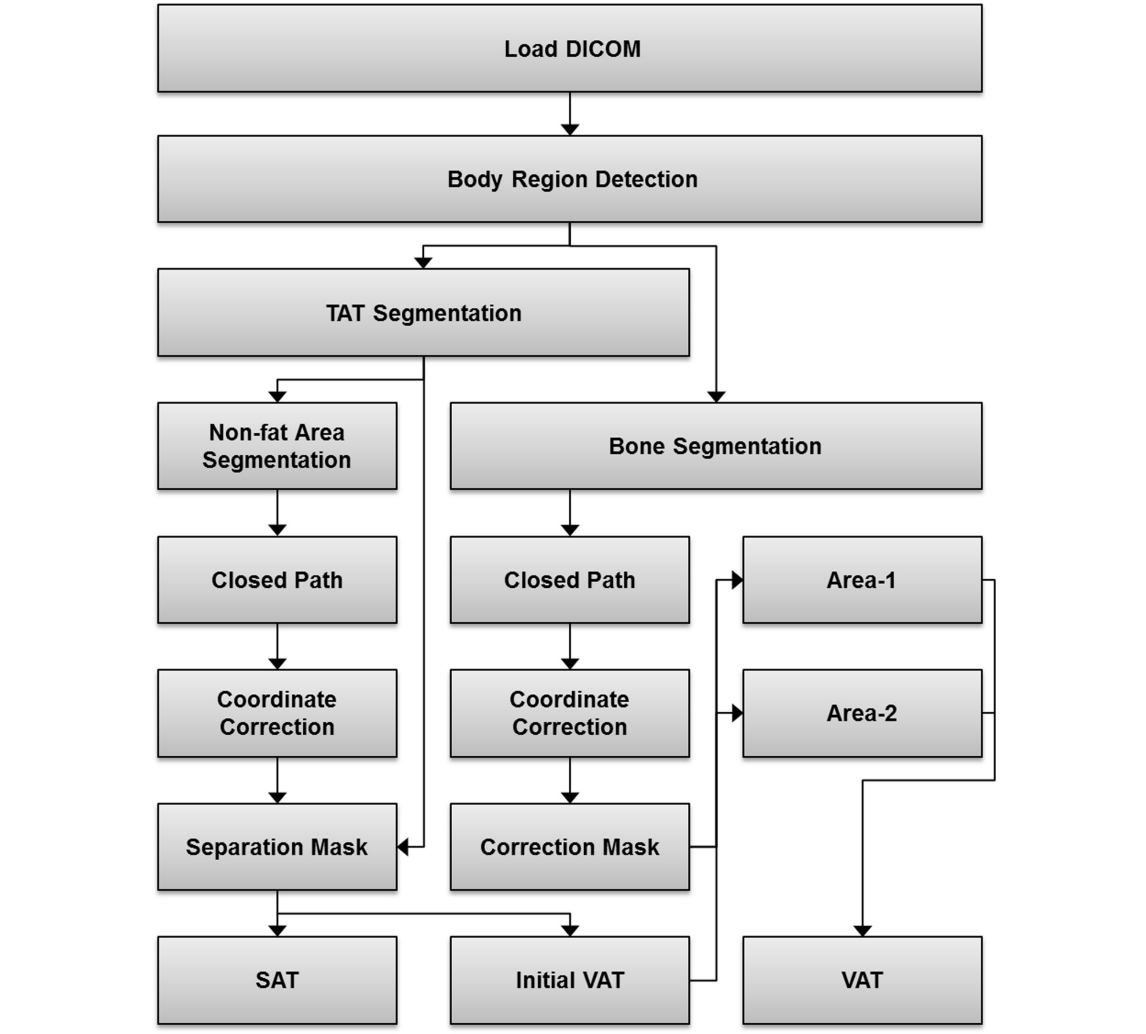
Algorithm flowchart.

The automated method of body fat proposed measurement described in this paper consists of the following three tasks: (1) pre-processing, (2) fat detection, and (3) post-processing. The complete flowchart of the algorithm is shown in Figure 1.

#### Preprocessing

CT images include various objects such as body, air, bed, and sheets. The efficiency of the algorithm was enhanced to perform the analysis only within the body region. The improved algorithm was applied to the pre-processing stage for the body-region detection. Unnecessary regions of the CT image were removed to prevent errors in detecting the body region. The body-region detection was performed using threshold and labeling techniques [16]. The air’s HU in CT images has attenuation lower than -1000 HU [17]. Knowing this, we eliminated the air region by setting thresholds and detected other regions by labeling. Subsequently, the body area was acquired by all the other labels, except the label with the widest area.

#### Fat Detection

AT in the abdominal area is sorted in SAT and VAT according to the location of AT and abdominal muscles; the interior of muscle is classified as SAT and the exterior as VAT. Thus, a separate mask was created based on the location of the abdominal muscles.

**Figure 2:**
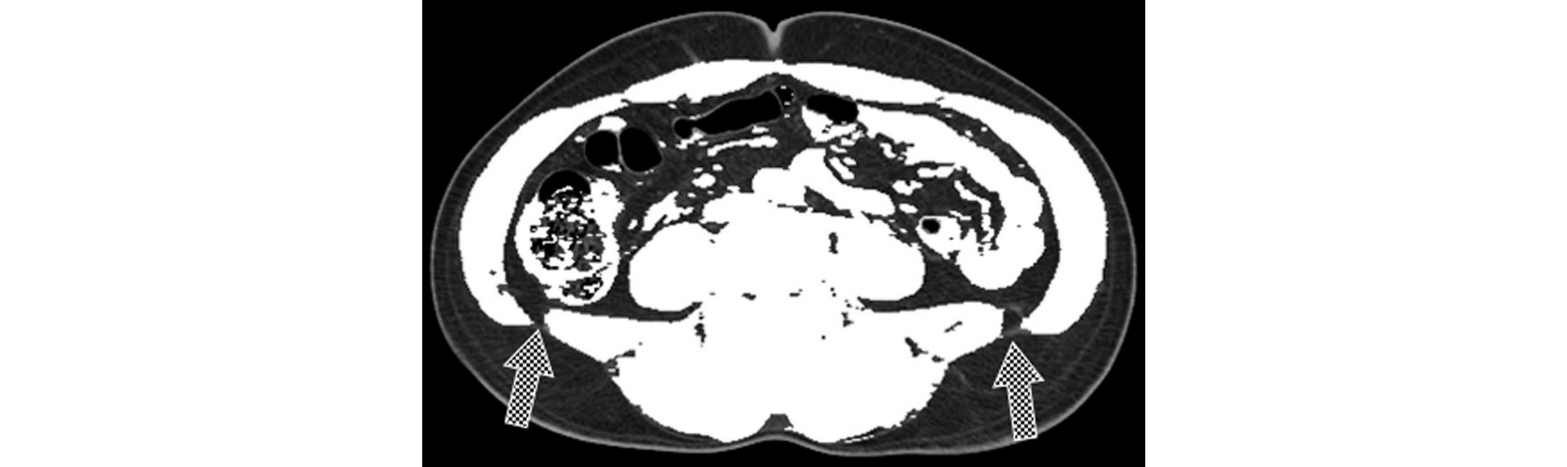
Result of the threshold application to the visceral region.

**Figure 3:**
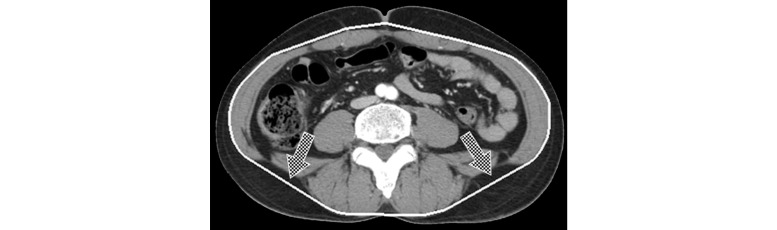
Result of the application of the Convex Hull algorithm and threshold.

**Figure 4:**
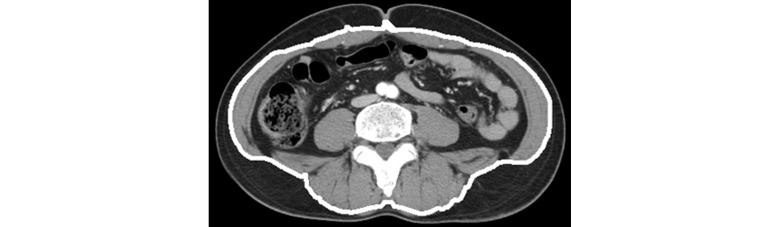
Result of the separation mask.

Creating a separation mask consists of three steps: (1) nonfat area segmentation; (2) closed path acquisition; (3) and correction of the closed path (Figures 2-4). Non-fat area detection is a process for finding the location of the muscle as the base of a separation mask and is performed by eliminating the abdominal fat region through thresholds. First, total adipose tissue (TAT) is obtained by setting a threshold at -30 to -190 HU, the attenuation range of fat in CT images, and the non-fat area is extracted by removing the TAT area from the body area [18]. After extraction, the skin area included in the area using opening, a morphology-based technique, is deleted. The result of this process shows that bones and organs inside the abdominal cavity as well as muscles are detected in the area (Figure 2). Muscle segmentation is omitted because bones and organs inside muscle don't affect a closed path, which is detected on the basis of the outermost muscle coordinates. In addition, the efficiency of the algorithm is increased by skipping the muscle segmentation process. Obtaining a closed path is the process for blocking the parts connected between SAT and VAT completely, such as the ones shown in Figure 2. The Convex Hull algorithm was used to detect the shortest closed path [19]. This algorithm determines the convex polygon of the minimum area included when a set of points or shapes are given. The Convex Hull results show the complete separation of organ areas without connecting the parts between SAT and VAT. However, errors can be generated as a closed path cannot be perfectly attached to muscle area when detecting a closed path (Figure 3). Because the errors can affect the quantitative results, each coordinate is corrected so that a closed path can be closer to the muscle area. Correction of errors is performed on all the coordinates that make up a closed path and the procedure is shown in Textbox 1.

**Procedure for the correction of errors on the coordinates that make up a closed path.**

1. Examine whether the closed path is in contact with the organ region relative to the *y* axis coordinates of the relevant closed path.
2. If it is not in contact, the relevant coordinate is deleted and moved to the location where it is in contact with the organ region.
3. After completing the examination of all closed path coordinates, randomly identify 1 coordinate as the starting point.
4. With the starting point as the standard, set the coordinate located at the shortest distance as the arrival point. After connecting it with a line, set the arrival point as the new starting point.
5. Repeat Stage 4 until it cannot be performed further.

**Figure 5:**
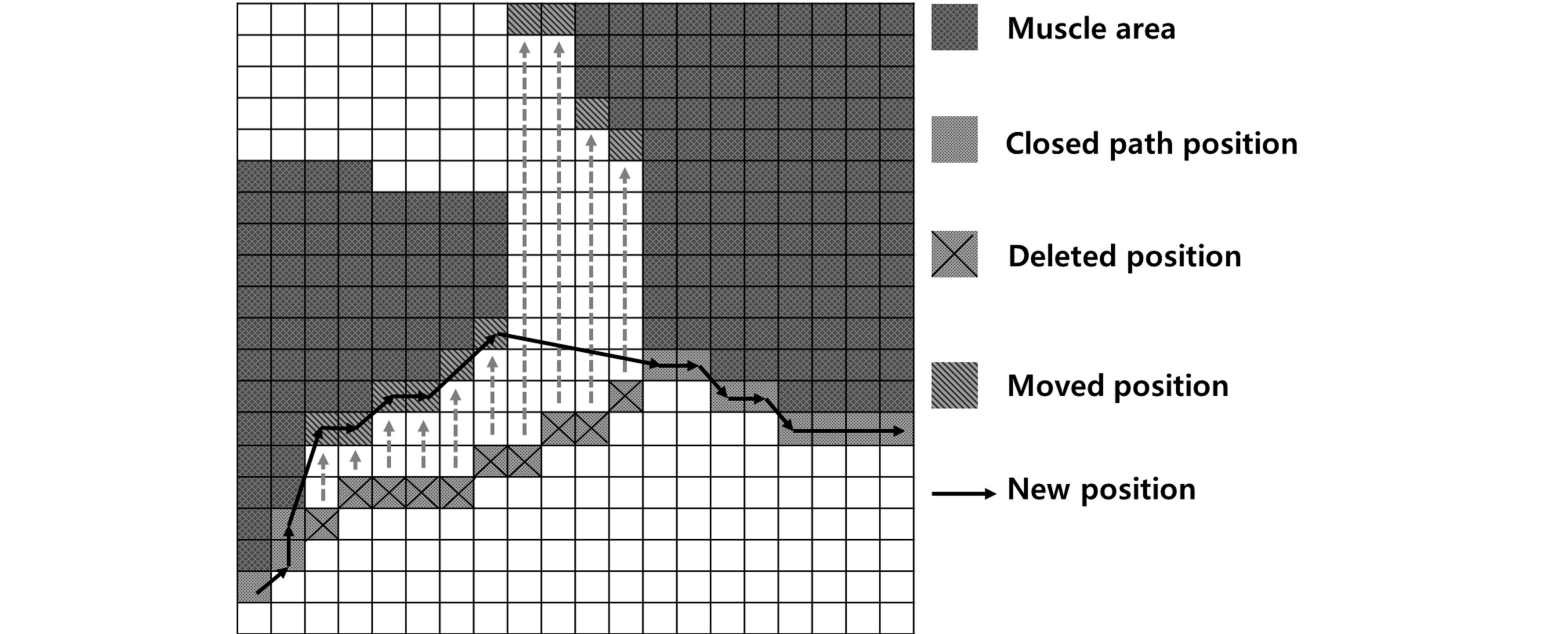
Process of coordinate correction.

The lines drawn through this method of coordinate correction become the new closed path of the organ region and produce the separation mask by filling in the interior of the closed path. The separation mask’s outline can be verified in Figure 4 and the entire coordinate correction process can be observed in Figure 5.

The produced separation mask sorts and detects for each region of SAT and VAT. SAT regions can be extracted by removing the separation mask from the TAT region detected by producing the separation mask. VAT regions can be detected through an “AND” operation between TAT and the mask regions.

#### Post-Processing

The fat component inside bones is detected as body fat in the process of detecting TAT, and it is included in the separation mask as bones are surrounded by muscles. Consequently, it is incorrectly detected as VAT. The spine, ribs, and pelvis surrounding the right and left abdominal cavity from the center of the back, and AT need be erased. In order to eliminate the false detection as VAT, a correction mask based on bones was included. Bones have a pixel range above 1000 HU in CT [17], and based on that, we set a seed point in the region estimated as bone and detected them using a 3D region growing algorithm [20]. Then, a correction mask was made by obtaining a closed path and applying a closed path-correction method to the extracted bone region in the same way as the separation mask. We attempted to apply different correction methods for each region after dividing the body into 2 regions based on bones. The regions are divided considering whether the right or left side of the abdominal cavity is surrounded by bones. The region that the ribs and pelvis are located in is surrounded on the back, right, and left sides of the abdominal cavity by bones. Therefore, it is necessary consider all of the sides of the abdominal cavity (Area 1). However, only the region of the back side that contains the spine needs to be imaged (Area 2). Considering these differences, the region detected as VAT outside a separation mask (back side, right and left side), as well as inside a separation mask for Area 1 were eliminated. The region recognized by the VAT in Area 2 in the back, outside of the separation mask, as well as inside the separation mask was removed. Any false-detected VAT was corrected using Area 1 and Area 2.

## Results

**Figure 6:**
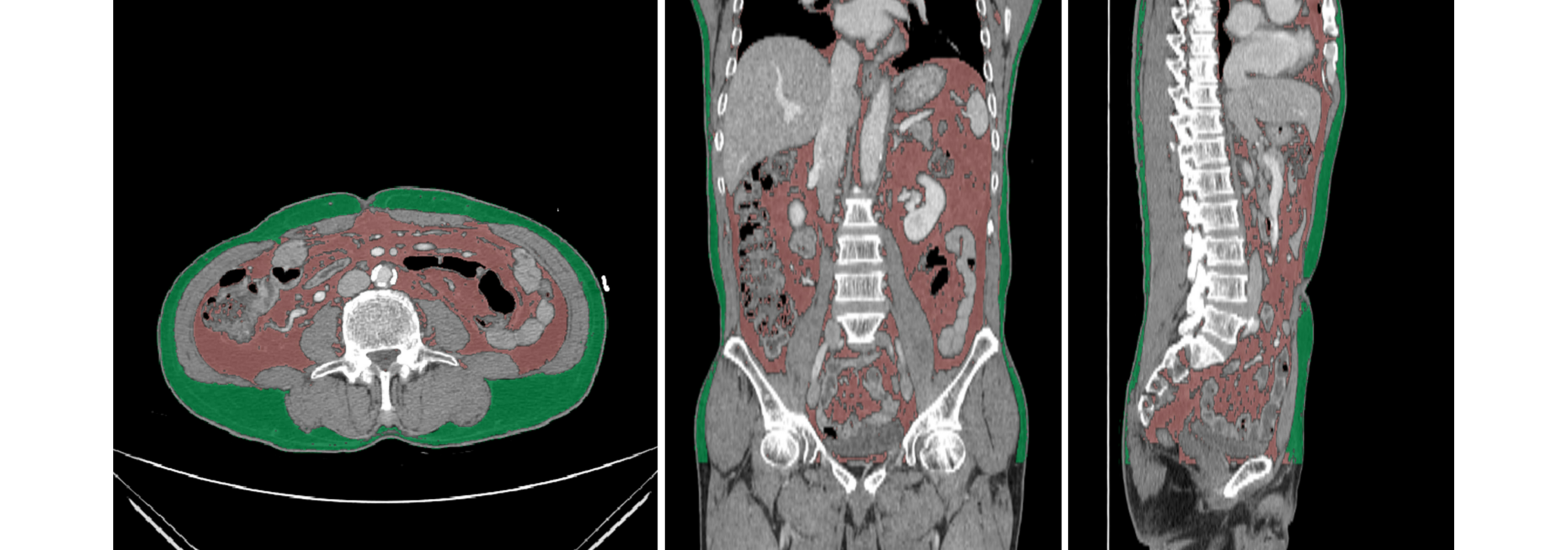
Automated segmentation and measurement results for SAT (green) and VAT (red).

In this study, we detected and measured the volume of TAT, SAT, and VAT for a total of 100 CT data using our automated method (Figure 6). As well, the automated measurement results (M_AUT_) were compared with the manual measurement results obtained by 2 experts (M_M1_, M_M2_). A comparative analysis was carried out between them in order to verify the technical accuracy and clinical reliability of the proposed method. The accuracy of the automated measurement method was evaluated through four kinds of conditional probability including sensitivity, specificity, accuracy, and Dice’s similarity coefficient (DSC). The automated measurement results and the manual measurement results were also compared through one-way analysis of variance (ANOVA) tests and Bland-Altman plots (see Multimedia Appendix 1), performing regression analysis and scatter plots (see Multimedia Appendix 2) to investigate the correlation between them. Moreover, the intraclass correlation coefficients (ICCs) between the results of both measurement methods were determined, and the reliability between the results examined. For the conditional probability test, true positive, false positive, true negative, and false negative were obtained by calculating, pixel-by-pixel, the position of VAT, SAT, and TAT detected by the automated and manual methods. The test results are shown in Table 1. The test results of M_M1_ indicate high precision, as the accuracy of M_M1_ for TAT, SAT, and VAT is 99.69%, 99.79%, and 99.79%, respectively. The DSC values for TAT, SAT, and VAT are 0.99, 0.98, and 0.97, respectively. Similar results were obtained for M_M2_, as the accuracy and DSC value for TAT is 99.62% and 0.98, 99.77% and 0.98 for SAT, and 99.74% and 0.97 for VAT..

**Table 1:** The conditional probability test between the automated and manual measurements.

		Sensitivity, %	Specificity, %	Accuracy, %	Dice similarity coefficient
**Automatic measurement (M**_**AUT**_ **) and manual measurement (M**_**M1**_ **)**					
	TAT	97.45	99.96	99.69	0.99
	SAT	97.24	99.98	99.79	0.98
	VAT	97.54	99.88	99.79	0.97
**Automatic measurement (M**_**AUT**_ **) and manual measurement (M**_**M2**_ **)**					
	TAT	97.39	99.89	99.62	0.98
	SAT	96.96	99.98	99.77	0.98
	VAT	97.87	99.81	99.74	0.97

The mean volume of TAT, SAT, and VAT measured by the automated method was 7913.79 mL (SD 2852.62 mL), 4620.38 mL (SD 1735.76 mL), and 3293.41 mL (SD 1497.11 mL), respectively. The mean volume for the same items measured by the manual method was 8021.56 mL (SD 2877.91 mL), 4750.01 mL (SD 1801.47 mL), and 3271.54 mL (SD 1469.16 mL) for M_M1_, and 7972.33 mL (SD 2889.43 mL), 4757.41 mL (SD 1822.06 mL), 3214.91 mL (SD 1473.27 mL) for M_M2_ (Table 2). The correlations are significant between the volumes of M_AUT_ and M_M1_ for TAT (*r*=.999, *P*<.001), SAT (*r*=.999, *P*<.001), and VAT(*r*=.999, *P*<.001) (Multimedia Appendices 2A-C). The correlations between the volumes of M_AUT_ and M_M2_ are also significant for TAT (*r*=.999, *P*<.001), SAT (*r*=.999, *P*<.001), and VAT (*r*=.999, *P*<.001) (Multimedia Appendices 2D-F).

**Table 2:** Comparison and verification between the results of the automated and manual measurements.

Item		Mean volume^a^ (SD)	*F*	*P* value^b^	ICC	*P* value^c^
**TAT**			.035	.965	.99	< .001
	M_AUT_	7913.79 (2852.62)				
	M_M1_	8021.56 (2877.91)				
	M_M2_	7972.33 (2889.43)				
**SAT**			.186	.830	.99	< .001
	M_AUT_	4620.38 (1735.76)				
	M_M1_	4750.01 (1801.47)				
	M_M2_	4757.41 (1822.06)				
**VAT**			.075	.928	.99	< .001
	M_AUT_	3293.41 (1497.11)				
	M_M1_	3271.54 (1469.16)	.			
	M_M2_	3214.91 (1473.27)				

^a^Mean volumes and SD measured in milliliter.

^b^ *P* value for ANOVA test.

^c^ *P* value for ICC.

One-way ANOVA test results for the volumes of M_AUT_, M_M1_, and M_M2_ revealed that no significant differences were found for TAT (*F*=.035, *P*=.965), SAT (*F*=.186, *P*=.830), and VAT (*F*=.075, *P*=.928; Table 2). Bland-Altman plots for the same items showed a good comparability as most volumes were within 1.96 standard deviations from the average position of the respective volume differences (Multimedia Appendices 1A-F). ICC values for the volumes of M_AUT_, M_M1_, and M_M2_ indicate high reliability for all the measuring items with .99 (CI 0.99-0.99, *P*<.001) for TAT, .99 (CI 0.97-0.99, *P*<.001) for SAT, and .99 (CI 0.98-0.99, *P*<.001) for VAT.

**Figure 7:**
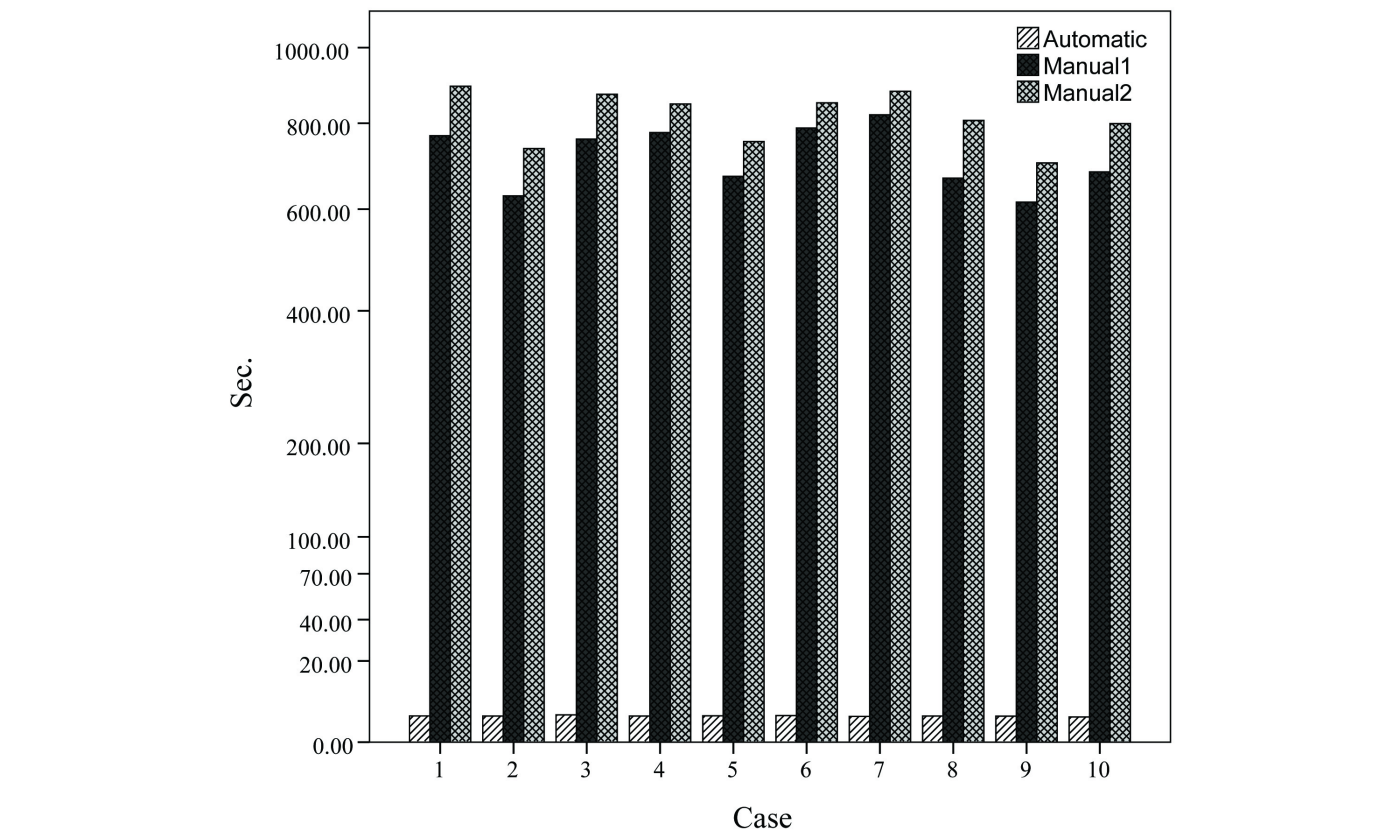
Comparison of the elapsed time required for measuring body fat within the range of the umbilical level (12 cm).

The elapsed time required to measure constant volume with both the automated and manual methods was compared to evaluate the usefulness of the proposed method. To do this, 20 data sets were randomly selected out of the 100 total, each containing a slice thickness of 5 mm. The elapsed time required to determine TAT, SAT, and VAT, within the range of the umbilical level (24 12 cm slices) by the automated and the manual method was measured. We found that the mean elapsed time required for manual measurements performed by 2 experts was 718.5 seconds (SD 72.7 seconds) for M_M1_ and 815.5 seconds (SD 65.8 seconds) for M_M2_, whereas the automated method (M_AUT_) required only 3.62 seconds (SD 0.1 seconds) (Figure 7).

##  Discussion

### Principal Findings

The previous AT measurement methods with CT were very tedious and time consuming. As such, many studies have attempted to find out the association between the indicators of obesity such as BMI, waist to hip ratio (WHR), and fat measurements with the typical CT used in clinic [21,22]. Although one CT has some association with diverse indicators of obesity, there’s a risk of error caused by measurements and evaluations of a 2-dimensional (2D) cross section of a 3-dimensional (3D) body. In addition, indicators such as BMI and WHR are not quantitative. Therefore, we proposed and verified a new method of separating and measuring SAT and VAT from the entire abdominal CT automatically by adopting an image processing technique. The test results of the proposed method demonstrate a high level of accuracy (99%) for TAT, SAT, and VAT compared to the results made manually (M_M1_ and M_M2_).

The results of our proposed method show a higher degree of accuracy compared with other reported studies (Table 3). Bandekar et al [12] applied the active shape model automatic fat methods based on fuzzy affinity and reported accuracies of 98.29% (SD 0.62%) and 97.66% (SD 0.98%) for SAT and VAT, respectively. Using a method of obtaining pixel information by a 3 degree radial movement from the body center to understand SAT and VAT on CT images, Zhao et al [13] reported accuracies of 99.35% for SAT and 98.46% for VAT. In another example, Kullberg et al [14] measured accuracies of 96% (SD 2.3%) for SAT and 90% (SD 6.5%) and VAT using a histogram based on MRI images in the umbilical level range and constructed AT and SAT masks to isolate each AT.

**Table 3:** Comparison of the automated measurement of AT proposed in this paper with previously published studies.

	Study			
	Bandekar et al [12]	Zhao et al [13]	Kullberg et al [14]	Proposed method
Modality	CT	CT	MRI	CT
Range	1 slice at umbilical level	1 slice at umbilical level	Volume of umbilical level	Volume of entire abdominal cavity
Number of data sets	40	9	17	100
Accuracy of SAT, % (SD)	98.29 (0.62)	99.35	96 (2.3)	99.78 (0.18)
Accuracy of VAT, % (SD)	97.66 (0.98)	98.46	90 (6.5)	99.76 (0.16)

With respect to measurement range, our proposed method can measure the entire abdominal cavity rather than the existing methods that are restricted to 2-dimensional levels or only the umbilical region. While the way of separating SAT and VAT using templates or masks is similar to other studies, in our proposed method, the separation mask is generated by minimizing the number of algorithms based on pixel value, approaching the anatomical shape. Furthermore, the analysis is a contributing factor for raising the level of accuracy.

The ANOVA test and ICC results demonstrate the clinical reliability of the proposed method (Table 2). The ANOVA test indicates that no noticeable differences were observed between the automated measurements and the manual measurements made by the 2 experts for TAT, SAT, and VAT. The ICC results show a very high level of reliability for TAT, SAT, and VAT (.99). However, we did find that the automated measurements had slightly smaller values than the manual measurement for SAT, while for VAT, the automated measures had the tendency to be slightly larger. A possible cause for the gap in SAT segmentation is because intermuscular fat or intramuscular fat near subcutaneous fat is included as part of subcutaneous fat, and it is accumulated during the manual measurement. The difference in VAT segmentation might be caused by the detection of some adipose tissue inside the bone as VAT during the correction process of the automated measurement. Although their impact on the overall segmentation results was negligible since the rate of error was low, an even higher degree of accuracy can be anticipated as the algorithms are improved in the future. The time-shortening effect of the automated method shows the time reduction to be about 200 fold compared to the manual method for a constant range (Figure 7). Based on this result, we can estimate that the time required to manually measure an abdominal CT consisting of 150 images with a 3 mm thickness to be approximately 80 min while the same can be done using the automated method in approximately 20 seconds. This supports the usefulness and power of the automated method. Although the proposed method uses CT for measurement, the structure of the algorithm is based on anatomical shape. Therefore, the potential exists to apply it to other imaging methods such as MRI.

### Conclusions

Measuring AT by the proposed CT method allows for a more quantitative and objective measurement result in a time efficient manner. As well, the simple measurements of VAT enabled by the new method will be very useful in evaluating visceral abdominal obesity. Furthermore, we expect that the proposed method will be more convenient and useful in clinical evaluations and studies on patients’ abdominal obesity levels.

## Multimedia Appendix 1

Bland-Altman plots.

## Multimedia Appendix 2

Scatter plots.

## Abbreviations

ANOVA: analysis of variance
AT: adipose tissue
BIA: bioelectrical impedance analysis
CT: computed tomography
DSC: Dice’s similarity coefficient
HU: Hounsfield unit
ICC: intraclass correlation coefficient
MRI: magnetic resonance image
SAT: subcutaneous adipose tissue
TAT: total adipose tissue
VAT: visceral adipose tissue
WHR: waist-to-hip ratio
