# Supplementary material for: Computerized Automated Quantification of Subcutaneous and Visceral Adipose Tissue From Computed Tomography Scans: Development and Validation Study
Source: JMIR Med Inform. 2016 Feb 4;4(1):e2. doi: 10.2196/medinform.4923 (PMC4759454; doi:10.2196/medinform.4923)

Bland–Altman Plots

(A) Plot comparing the automated measurement (MAUT) and the manual measurements (MM1) results of TAT.

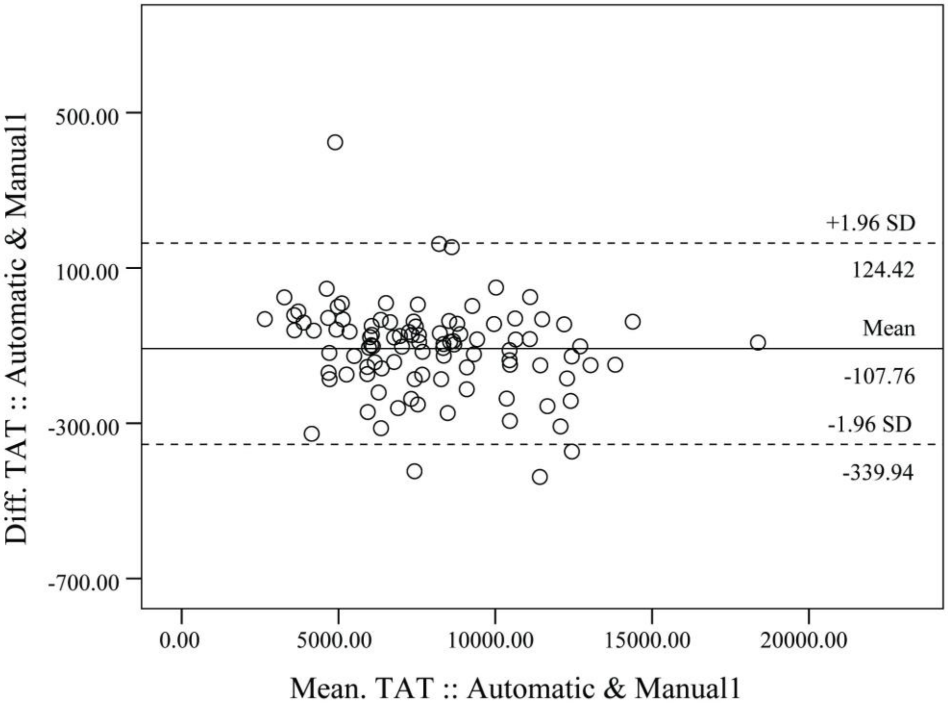

(B) Plot comparing the automated measurement (MAUT) and the manual measurements (MM1) results of SAT.

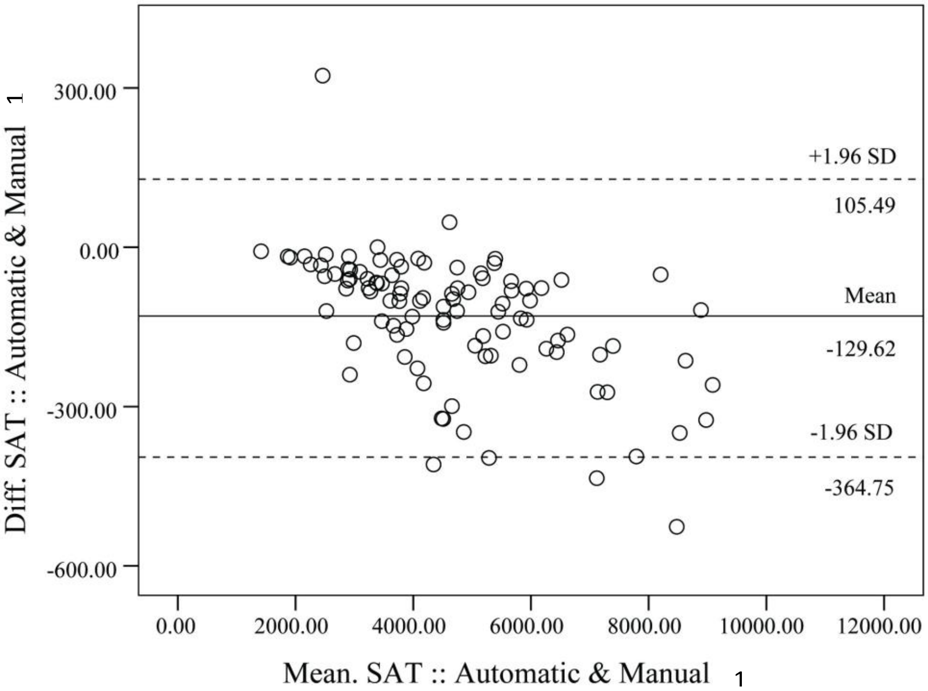

(C) Plot comparing the automated measurement (MAUT) and the manual measurements (MM1) results of VAT.

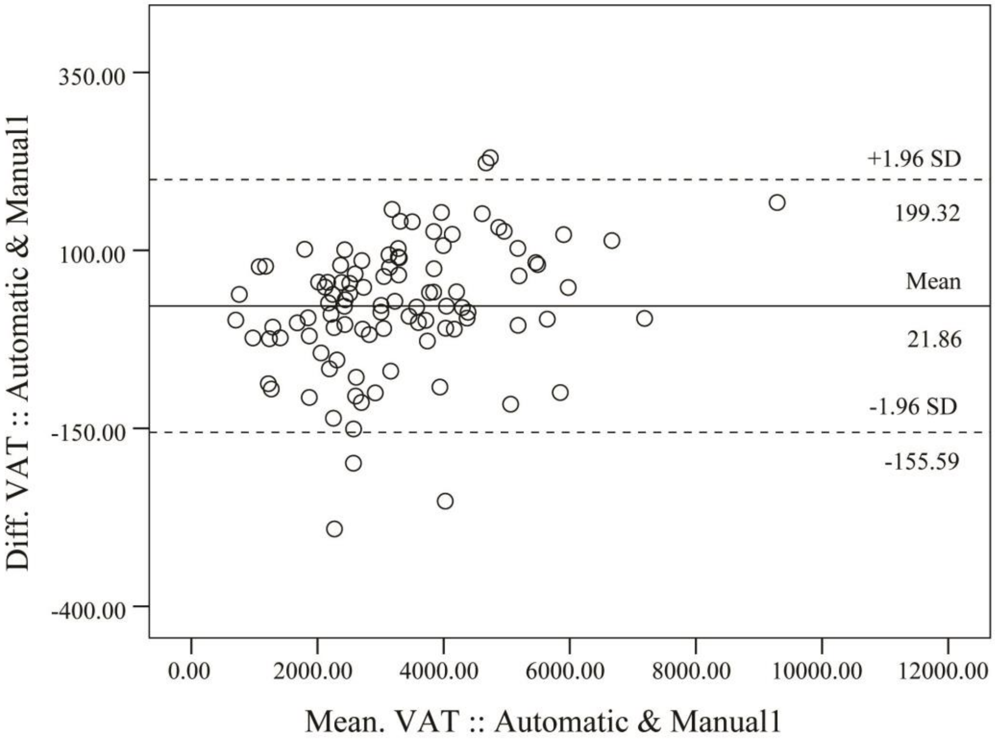

(D) Plot comparing the automated measurement (MAUT) and the manual measurements (MM2) results of TAT.

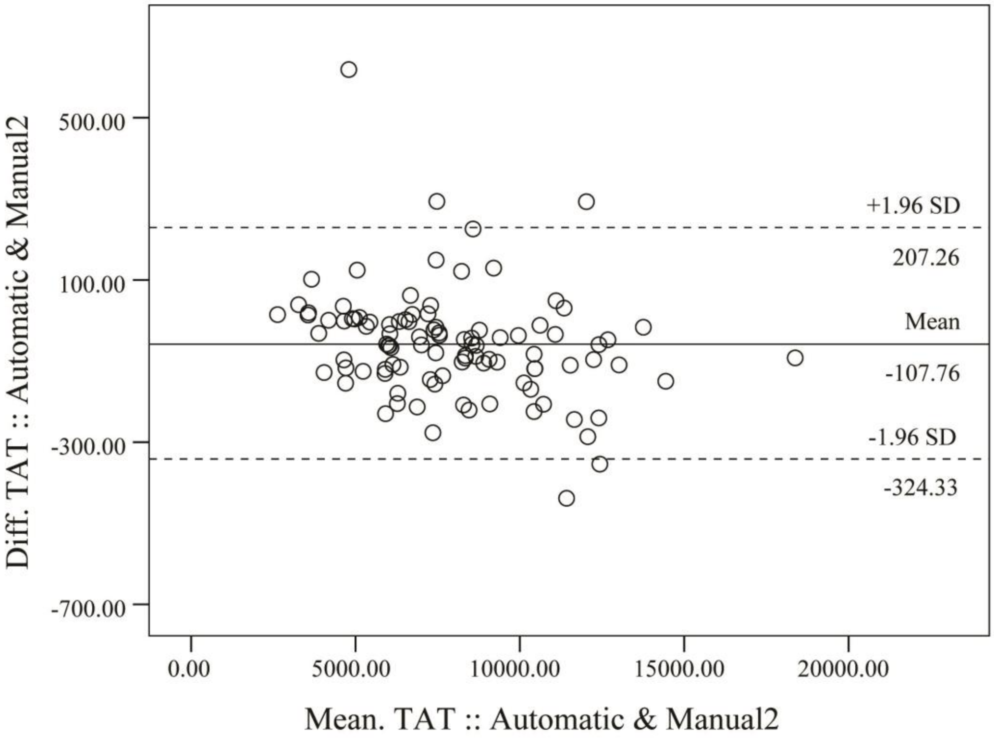

(E) Plot comparing the automated measurement (MAUT) and the manual measurements (MM2) results of SAT.

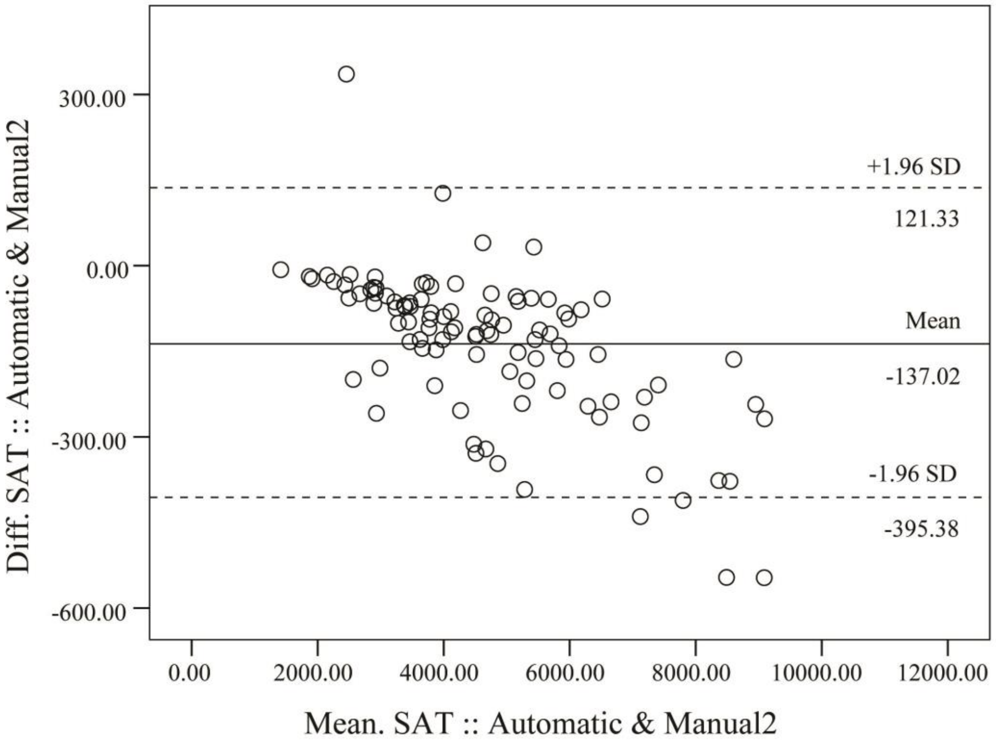

(F) Plot comparing the automated measurement (MAUT) and the manual measurements (MM2) results of VAT.

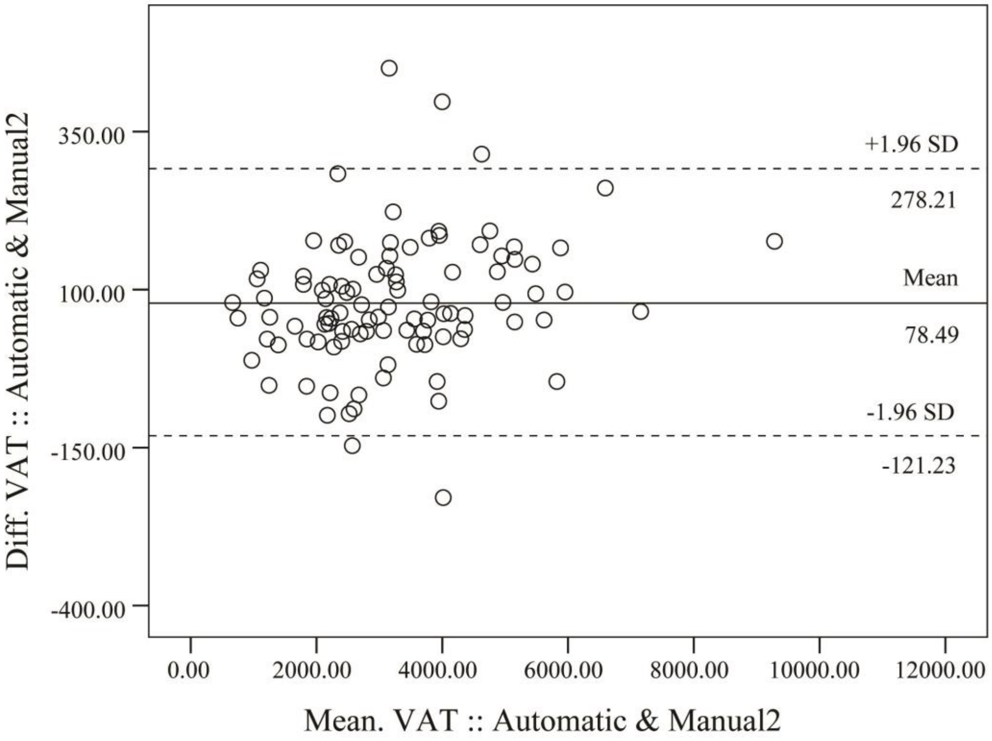

Supplement: Supplementary file 1 [file medinform_v4i1e2_app1.pdf]
