# Supplementary material for: Computerized Automated Quantification of Subcutaneous and Visceral Adipose Tissue From Computed Tomography Scans: Development and Validation Study
Source: JMIR Med Inform. 2016 Feb 4;4(1):e2. doi: 10.2196/medinform.4923 (PMC4759454; doi:10.2196/medinform.4923)

Scatter Plots

(A) Plot for the correlation between the automated measurement (MAUT) and the manual measurements (MM1) results of TAT.

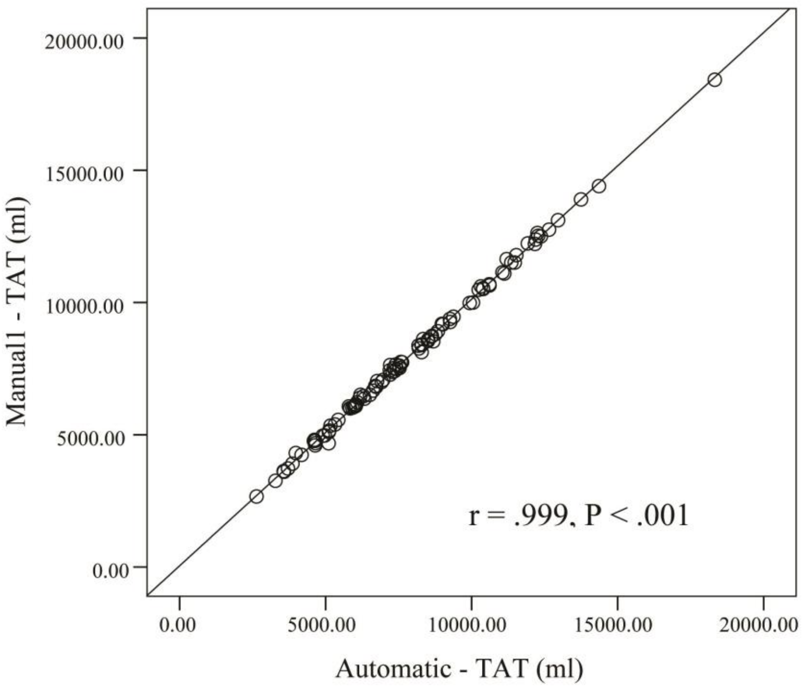

(B) Plot for the correlation between the automated measurement (MAUT) and the manual measurements (MM1) results of SAT.

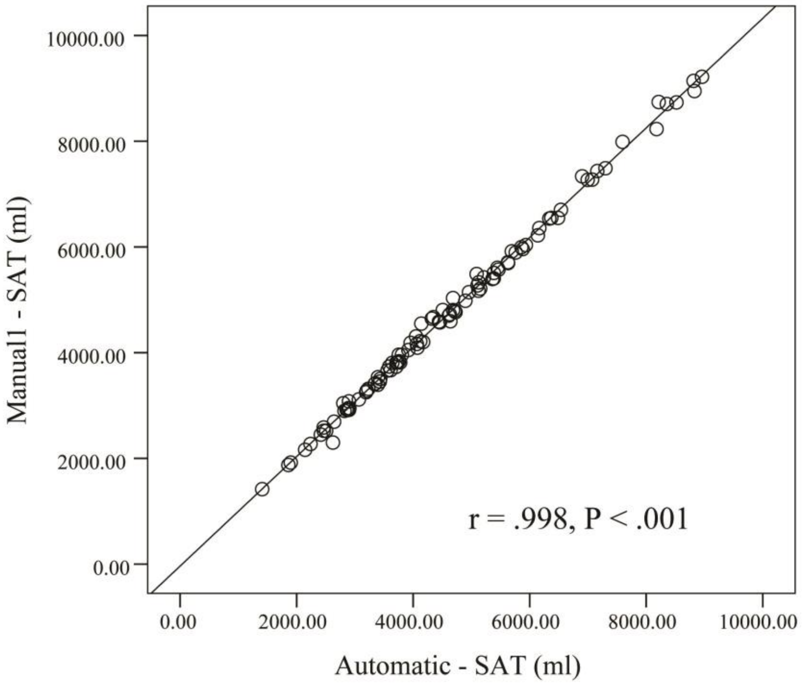

(C) Plot for the correlation between the automated measurement (MAUT) and the manual measurements (MM1) results of VAT.

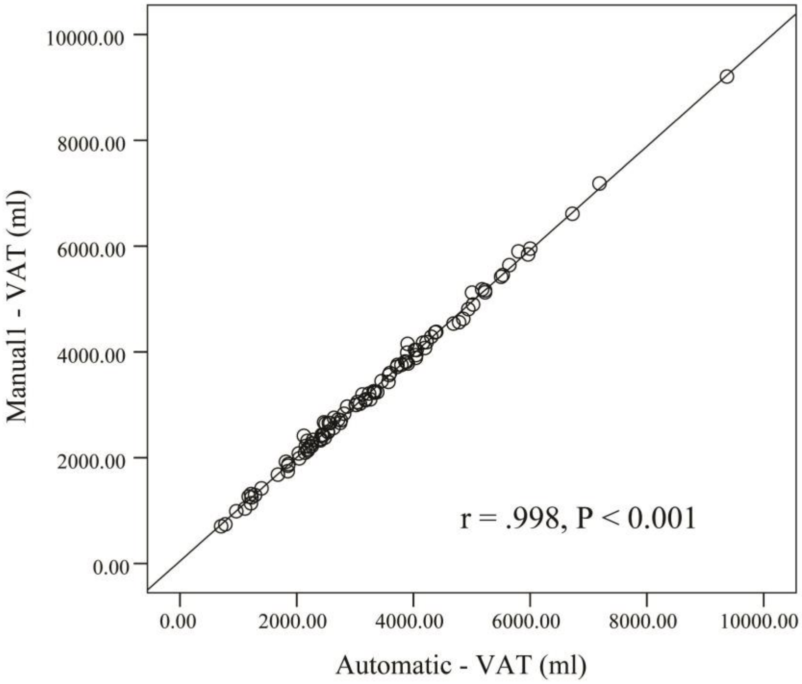

(D) Plot for the correlation between the automated measurement (MAUT) and the manual measurements (MM2) results of SAT.

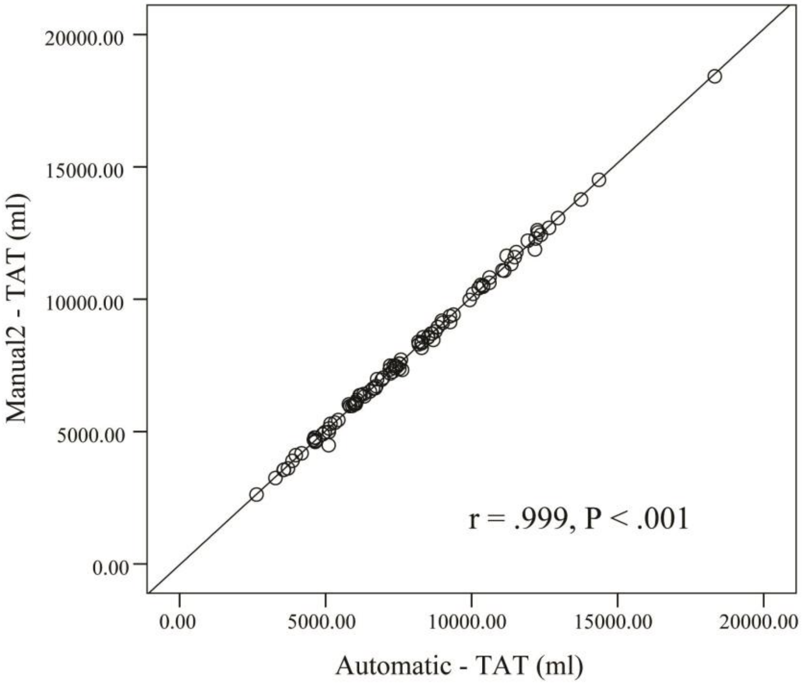

(E) Plot for the correlation between the automated measurement (MAUT) and the manual measurements (MM2) results of TAT.

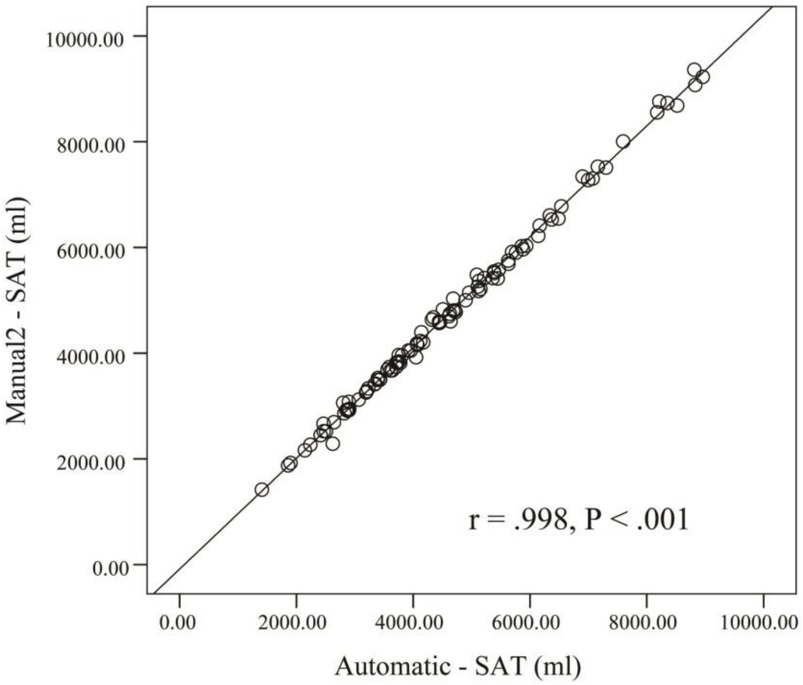

(F) Plot for the correlation between the automated measurement (MAUT) and the manual measurements (MM2) results of VAT.

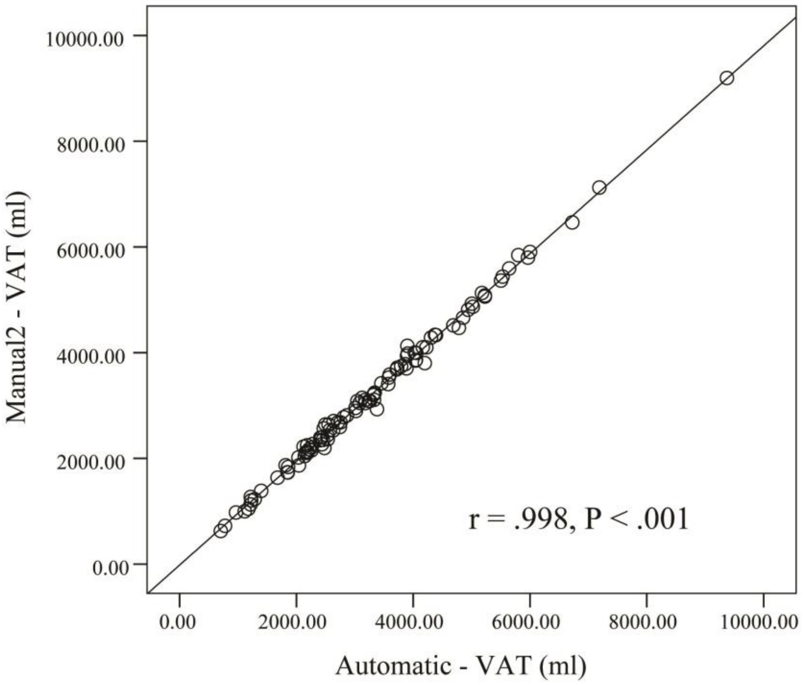

Supplement: Supplementary file 2 [file medinform_v4i1e2_app2.pdf]
